# Delineation of Chondroid Lipoma: An Immunohistochemical and Molecular Biological Analysis

**DOI:** 10.1155/2011/638403

**Published:** 2011-04-20

**Authors:** Ronald S. A. de Vreeze, Frits van Coevorden, Lucie Boerrigter, Petra M. Nederlof, Rick L. Haas, Johannes Bras, Andreas Rosenwald, Thomas Mentzel, Daphne de Jong

**Affiliations:** ^1^Department of Surgical Oncology, The Netherlands Cancer Institute, Antoni van Leeuwenhoek Hospital, 1066 CX, Amsterdam, The Netherlands; ^2^Department of Pathology, The Netherlands Cancer Institute, Antoni van Leeuwenhoek Hospital, 1066 CX, Amsterdam, The Netherlands; ^3^Department of Radiation Oncology, The Netherlands Cancer Institute, Antoni van Leeuwenhoek Hospital, 1066 CX, Amsterdam, The Netherlands; ^4^Department of Pathology, Academic Medical Centre, 1105 AZ, Amsterdam, The Netherlands; ^5^Department of Pathology, University of Wuerzburg, 97074 Wuerzburg, Germany; ^6^Department of Dermatopathology, Bodensee, D-88048 Friedrichshafen, Germany

## Abstract

*Aims*. Chondroid lipoma (CL) is a benign tumor that mimics a variety of soft tissue tumors and is characterized by translocation *t*(11;16). Here, we analyze CL and its histological mimics. *Methods*. CL (*n* = 4) was compared to a variety of histological mimics (*n* = 83) for morphological aspects and immunohistochemical features including cyclinD1(*CCND1*). Using FISH analysis, *CCND1* and *FUS* were investigated as potential translocation partners. *Results*. All CLs were strongly positive for *CCND1*. One of 4 myoepitheliomas, *CCND1*, was positive. In well-differentiated lipomatous tumors and in chondrosarcomas, *CCND1* was frequently expressed, but all myxoid liposarcomas were negative. FISH analysis did not give support for direct involvement of *CCND1* and *FUS* as translocation partners. *Conclusions*. Chondroid lipoma is extremely rare and has several and more prevalent histological mimics. The differential diagnosis of chondroid lipomas can be unraveled using immunohistochemical and molecular support.

## 1. Introduction

Lipomatous lesions show a broad morphological spectrum and clinically range from benign to highly malignant diseases. Over the last few years, studies focusing on lipomatous tumors have led to the delineation of new variants of lipomatous proliferations as well as to the introduction of new concepts, mainly as a result of the fruitful interactions between molecular genetics and pathology [1–4]. As a result, chondroid lipoma has been described and considered a benign tumor of soft tissue that may mimic a variety of soft tissue tumors [1, 5–7].

At gross examination, chondroid lipoma resembles lipoma, presenting as a solitary, slowly growing mass that is located either within skeletal muscle, muscle fascia, or in the deep subcutis. The main cytological features consist of clustered, variably mature, multivacuolated hibernoma-like cells enmeshed in a capillary plexus, in a background of chondromyxoid material. This tumor may show histologic features resembling myoepithelioma, myxoid liposarcoma, extraskeletal myxoid chondrosarcoma, hibernoma, and other lipomatous or chondroid neoplasms, resulting in diagnostic and consequently therapeutic dilemmas [8]. 

Cytogenetic data on a few cases of chondroid lipoma are available and show a balanced translocation *t*(11;16) (q13-p12) [9–11]. The typical and recurrent involvement of 11q13 has also been described in other classes of lipomatous tumors such as ordinary lipoma and hibernoma, but not in association with 16p12-13. Several genes at the breakpoint regions may be relevant candidate genes, and recently MKL/myocardin-like 2 (MKL2) has been implicated. Recent findings show that cyclinD1 (*CCND1*) is not only involved in cell cycle regulation, but also in the regulation of cellular metabolism, cellular migration, and especially fat cell differentiation, making this a relevant candidate gene [12]. Another candidate fusion gene is the *FUS* gene located on chromosome 16p11. This gene is involved in one of the typical mimics of chondroid lipomas: myxoid liposarcoma. Although *FUS* is located at a different chromosomal location (16p11 versus 16p12) and therefore involvement in chondroid lipomas is not highly likely, it has not been properly investigated.

Here, we describe a histopathological, immunohistochemical and fluorescence in situ hybridization analysis in a series of chondroid lipomas and histological mimics.

## 2. Methods

To retrieve all cases diagnosed as chondroid lipomas in the Netherlands between 1997 and 2007, a search was performed in the Dutch nationwide pathology registry database (Pathologic Anatomic National Automated Archive, PALGA). The PALGA database contains all reports of potential cases and anonymous patient characteristics such as age, gender, conclusions, and coded summaries of all pathology reports in the Netherlands since 1992. Potential cases were retrieved and corresponding formalin fixed paraffin embedded tissue blocks were collected from the original pathology laboratories. Eleven cases were reviewed on hematoxylin and eosin-stained slides with additional immunohistochemical and molecular data if needed (RdV, DdJ and JB); consensus was obtained at the multiheaded microscope. Two additional cases of chondroid lipoma were retrieved from the files of the Department of Dermatology, Bodensee, Friedrichshafen (Dr. T. Mentzel) and also included for further study. Classification of all biopsy and resection material was performed according to the WHO classification for soft tissue tumors [13]. Four cases were diagnosed as chondroid lipoma four cases as myoepithelioma and selected for further study. The remaining cases were diagnosed as myxoid liposarcoma (*n* = 1), lipoma (*n* = 1), and hibernoma (*n* = 1), chondrolipoma (*n* = 2). 

Specifically, to further investigate the role of *CCND1 *in the spectrum of lipomatous tumors, 21 lipomas, 28 well-differentiated liposarcomas, 18 myxoid liposarcomas, and 10 chondrosarcomas, both extraskeletal myxoid chondrosarcomas and primary chondrosarcomas of bone with extension into the soft tissue that were diagnosed in the same period, were randomly selected from the files of the Netherlands Cancer Institute to complete the morphological spectrum.

### 2.1. Immunohistochemical Analysis

Immunohistochemical staining was performed according to standard methods. In brief, after a pretreatment of citrate-based microwave antigen retrieval, the sections were incubated with the following antibodies overnight at 4°C without pretreatment: *CCND1* antibody (SP-4 AB-5 Labvision, Fremont, USA) dilution 1 : 100, *CD34* antibody (QBEND Labvision, Fremont, USA) dilution 1 : 3000, *CD68* antibody (KP1 DAKO Glostrup Denmark) dilution 1 : 50000, S100 antibody (polyclonal DAKO Glostrup Denmark) dilution 1 : 6000, Pan Keratin antibody (MNF116 + LP34 DAKO Glostrup Denmark) dilution 1 : 1600, *P63* antibody (MNF116 + LP34 LabVission Fremont USA) dilution 1 : 5000, *SMA* antibody (1A4 Zymed Carlsbad USA) dilution 1 : 5, vimentin antibody (3B4 DAKO Glostrup Denmark) dilution 1 : 400 and visualized with diaminobenzidine. The percentage of tumor cells with nuclear staining was assessed semiquantitatively. Staining intensity was ranked in three levels (positive, focal positive, and negative). Immunohistochemical staining of all slides were scored by two observers (DdJ and RdV). Slides could only be scored negative if positive internal controls were present. In cases of discrepancies or equivocal interpretations, consensus was obtained at the multiheaded microscope.

### 2.2. Fluorescence in Situ Hybridization

After confirming the most histologically typical areas using hematoxylin and eosin stained sections, dual-colour fluorescence in situ hybridization assay was performed according to standard methods on 5-*μ*m-thick tissue sections of formalin fixed paraffin embedded specimens. After dewaxing, hydration, and pretreatment (DAKO Glostrup Denmark) at 95°C for 10 min, a protease digestion was performed in for 15 min at 37°C. Incubation was performed according to the dual-color break-apart principle with two differently labeled probes flanking the gene of interest. For *CCND1*, one Texas Red-labeled DNA probe (*CCND1-Upstream*) covering 163 kb centromeric to the *CCND1* breakpoint cluster region and one fluorescein-labeled DNA probe (*CCND1-Downstream*) covering 644 kb telomeric to the *CCND1* breakpoint cluster region were cohybridized (DAKO Glostrup Denmark). For analysis of *FUS* (16p11), one Spectrum Green labeled probe distal to the *FUS *gene and one Spectrum Orange labeled probe proximally from the *FUS* gene were used. Slides were incubated at 37°C for 48 h in a humidified chamber. After stringent washing at 72°C for 2 min and counterstaining, fluorescent signals were scored using a Nikon Microphot-SA fluorescence microscope with appropriate filters, and the resulting images were captured using a charge-coupled-device camera. Fifty to 60 evaluable nuclei were counted by two different individuals (PN and RdV), and the percentages of single and fused signals were calculated. A positive result was defined as the presence of split signals in more than 10% of the cells when the distance between the flanking signals was three times the estimated signal diameter. In case of two single color pairs in more than 90% of the cells, cells were regarded negative for translocation.

## 3. Results

### 3.1. Patient Selection

In the initial PALGA and the Bodensee, Friedrichshafen (Dr. T. Mentzel) selection, 15 patients were retrieved. Of these, 2 cases were excluded because representative material could not be obtained. At review, four lesions were considered true chondroid lipomas. Further, four myoepitheliomas: one myxoid liposarcoma, one lipoma, one hibernoma, and two chondro lipomas were diagnosed (Table 1). The four chondroid lipomas and four myoepitheliomas were further analyzed and compared to 83 mimics randomly selected cases collected at the Netherlands Cancer Institute as described above.

### 3.2. Histopathological Analysis

**Figure 1:**
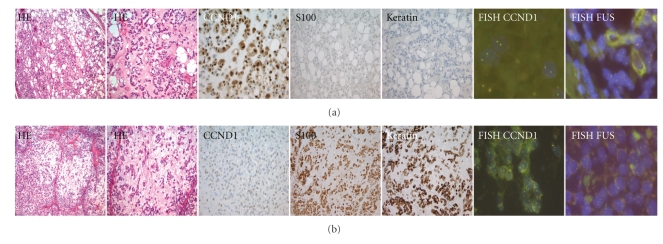
(a) Chondroid lipoma and (b) myoepithelioma.

Chondroid lipomas (*n* = 4) showed fibrous capsule and were dominated by a mature lipomatous proliferation with sheets, clusters, and nests of cells with eosinophilic, vacuolated cytoplasm in an eosinophilic cartilagenous matrix. The extracellular myxohyaline matrix showed a cartilagenous appearance, and the vascularisation was rich (Figure 1). The eosinophilic and vacuolated tumor cells were arranged in sheets, clusters, and cords and contain irregular, hyperchromatic nuclei with inconspicuous nucleoli; some of the vacuolated cells are indistinguishable from lipoblasts. Mitotic activity was absent.

### 3.3. Immunohistochemical Analysis

All (4/4) myoepitheliomas stained positive for keratin, whereas 1/4 chondroid lipoma stained focally positive and 3/4 stained negative. Immunohistochemical staining with S100 showed 1/4 positive and 2/4 focal positive lesions in chondroid lipoma and 3/4 positive and 1/4 focal positive myoepithelioma. Immunohistochemical staining of chondroid lipomas and myoepithelioma for vimentin, *SMA, CD34, CD68*, and *P53* was not distinctive. 

Detailed immunohistochemical results are listed in Table 2 and Figure 1.

### 3.4. CCND1 and FUS as Candidate Genes for Translocation in Chondroid Lipoma

In all chondroid lipomas, both the obvious lipogenic cells and the eosinophilic tumor cells were immunohistochemically uniformly positive for *CCND1*, whereas 1/4 myoepitheloma lesion stained positive for *CCND1*, and 3/10 chondrosarcomas stained positive while 3/10 were focal positive and 4/10 were negative. The lipomas and well-differentiated liposarcomas that were immunohistochemically analyzed for *CCND1* showed scattered positivity in a majority of cases 32/49 (65%), whereas 16/49 (33%) stained negative for *CCND1 *and 1/49 stained positive. None of the 18 myxoid liposarcomas showed immunohistochemically *CCND1 *expression. Therefore, *CCND1* could be used for distinction between myxoid liposarcoma and chondroid lipoma: 4/4 (100%) positivity in chondroid lipoma and 0/18 (0%) positivity in myxoid liposarcoma. 

By using fluorescence in situ hybridization for *CCND1 *and *FUS*, respectively, no breaks in these genes could be detected in chondroid lipoma or in myoepithelioma.

## 4. Discussion

This study shows that true chondroid lipomas are extremely rare soft tissue tumors. The fact that in a Dutch nationwide search in a 10-year period by PALGA only two unequivocal cases were diagnosed that were retrieved within a spectrum of mimics underlines the rarity of the diagnosis and shows that awareness of the characteristics of chondroid lipoma is particularly important in reaching a chondroid lipoma diagnosis.

This study furthermore shows that the histological mimics of chondroid lipomas as described in the literature can be distinguished by means of immunohistochemical analysis. Especially immunohistochemistry for *CCND1* and FISH analysis for the specific translocations may be supportive to discriminate between myxoid liposarcoma and chondroid lipoma. Although some apparent histologic hallmarks of chondroid lipomas can be readily recognized such as nests and cords of uni- and multivacuolated cells within a prominent myxohyaline to chondroid matrix, the immunohistochemical marker pattern is very helpful. Myoepithelioma of the soft tissues is an important mimic that expresses, in contrast to chondroid lipoma, the epithelial markers keratin as well as *S100 *protein. Well-differentiated liposarcoma with myxoid changes may be a mimic that may be recognized on the basis of clinical setting, morphology as well as by the specific genetic changes. Also chondroid lipoma may be characterized by a specific translocation *t*(11;16). Recently, MKL/myocardin-like 2 (MKL2) and C11orf95 (chromosome 11 open reading frame 95) were identified as translocation partners in 3 cases of chondroid lipoma. Although the extent of possible variant translocations may not be clear yet, this finding provides an important addition for further support in differential diagnostic problems [11, 14]. In the present study, all cases of chondroid lipomas showed high immunohistochemical expression of *CCND1. *Since the *CCND1* gene is located on 11q13, this makes it an attractive candidate gene for the *t*(11;16) translocation in chondroid lipoma. Based on a spilt apart FISH assay, *CCND1* did not show rearrangement, however. This indicates that the breakpoint is most probably not located in the *CCND1* region and does not give support for involvement of this gene in the oncogenesis of chondroid lipoma, despite expression of the protein. Indeed, *CCND1* is expressed broadly in several types of well-differentiated lipomatous tumors, such as lipoma and well-differentiated liposarcoma and also in tumors with chondroid differentiation, including extraskeletal myxoid chondrosarcoma and primary chondrosarcoma of bone in support of this notion. As expected, *FUS* indeed was shown not be involved in the translocation.

## 5. Conclusion

Chondroid lipoma is extremely rare and has several and more prevalent histological mimics. The differential diagnosis of chondroid lipomas can be unraveled using immunohistochemical and molecular support. Although chondroid lipoma shows high expression of *CCND1*, this expression should not be regarded as deregulated and there is no support that *CCND1 *is directly involved as a translocation partner in the characteristic *t*(11;16).

##  Conflict of Interests

The authors declared that there is no conflict of interests.

## Figures and Tables

**Table 1:** Patient and tumor characteristics.

Patient characteristics	Chondroid lipoma	Myo epithelioma	lipoma	Well-differentiated liposarcoma	Myxoid liposarcoma	Chondrosarcoma
Male/female	1/3	3/1	11/10	13/15	10/8	8/2
Median age at Diagnose, yrs (range)	36 (32–75)	56 (48–64)	49 (21–69)	62 (28–80)	43 (26–70)	54 (29–90)
Mean tumor circumference (range)	3 (2–10)	8 (2–20)	5 (1–18)	13,5 (2–30)	14 (4–30)	6 (2–13)

**Table 2:** Immunohistochemical and fluorescence in situ hybridization results.

Histology	IHC	IHC	IHC	IHC	IHC	IHC	IHC	IHC	FISH	FISH
	*CCND1* (%)	CD34	CD68	S100	keratin	P63	SMA	vimentin	Split apart *CCND1 *	Split apart *FUS *
Chondroid lipoma (*n* = 4)										
Positive	4	0	0	1	0	0	0	3	0	0
Focal positive	0	0	1	2	2	0	0	0	n.a.	n.a.
Negative	0	4	3	1	2	4	4	1	4/4*	4/4*

Myoepithelioma (*n* = 4)										
Positive	1	0	0	3	3	0	0	4	0	0
Focal positive	1	0	1	1	1	1	0	0	n.a.	n.a.
Negative	2	4	3	0	0	3	4	0	4/4*	3/3*

Lipoma (*n* = 21)										
Positive	0	—	—	—	—	—	—	—	—	—
Focal positive	16 (76)	—	—	—	—	—	—	—	—	—
Negative	5 (24)	—	—	—	—	—	—	—	—	—

Well differentiated liposarcoma (*n* = 28)										
Positive	1 (4)	—	—	—	—	—	—	—	—	—
Focal positive	16 (57)	—	—	—	—	—	—	—	—	—
Negative	11 (39)	—	—	—	—	—	—	—	—	—

Myxoid liposarcoma (*n* = 18)										
Positive	0	—	—	—	—	—	—	—	—	—
Focal positive	0	—	—	—	—	—	—	—	—	—
Negative	18 (100)	—	—	—	—	—	—	—	—	—

Chondrosarcoma (*n* = 10)										
Positive	3 (30)	—	—	—	—	—	—	—	—	—
Focal positive	3 (30)	—	—	—	—	—	—	—	—	—
Negative	4 (40)	—	—	—	—	—	—	—	—	—

*There were no translocations observed.

n.a.: not applicable.

IHC: immunohistochemical.

## References

[1] Meis JM, Enzinger FM (1993). Chondroid lipoma: a unique tumor simulating liposarcoma and myxoid chondrosarcoma. *American Journal of Surgical Pathology*.

[2] Katzer B (1989). Histopathology of rare chondroosteoblastic metaplasia in benign lipomas. *Pathology Research and Practice*.

[3] Thomson TA, Horsman D, Bainbridge TC (1999). Cytogenetic and cytologic features of chondroid lipoma of soft tissue. *Modern Pathology*.

[4] Nielsen GP, O’Connell JX, Dickersin GR, Rosenberg AE (1995). Chondroid lipoma, a tumor of white fat cells: a brief report of two cases with ultrastructural analysis. *American Journal of Surgical Pathology*.

[5] Gisselsson D, Domanski HA, Höglund M (1999). Unique cytological features and chromosome aberrations in chondroid lipoma: a case report based on fine-needle aspiration cytology, histopathology, electron microscopy, chromosome banding, and molecular cytogenetics. *American Journal of Surgical Pathology*.

[6] Hyzy MD, Hogendoorn PCW, Bloem JL, de Schepper AM (2006). Chondroid lipoma: findings on radiography and MRI (2006:7b). *European Radiology*.

[7] Green RAR, Cannon SR, Flanagan AM (2004). Chondroid lipoma: correlation of imaging findings and histopathology of an unusual benign lesion. *Skeletal Radiology*.

[8] Kindblom LG, Meis-Kindblom JM (1995). Chondroid lipoma: an ultrastructural and immunohistochemical analysis with further observations regarding its differentiation. *Human Pathology*.

[9] Thomson TA, Horsman D, Bainbridge TC (1999). Cytogenetic and cytologic features of chondroid lipoma of soft tissue. *Modern Pathology*.

[10] Ballaux F, Debiec-Rychter M, De Wever I, Sciot R (2004). Chondroid lipoma is characterized by t(11;16)(q13;p12-13). *Virchows Archiv*.

[11] Huang D, Sumegi J, Dal CP (2010). C11orf95-MKL2 is the resulting fusion oncogene of t(11;16)(q13;p13) in chondroid lipoma. *Genes Chromosomes and Cancer*.

[12] Fu M, Wang C, Li Z, Sakamaki T, Pestell RG (2004). Minireview: cyclin D1: normal and abnormal functions. *Endocrinology*.

[13] Fletcher CD (2002). *Pathology & Genetics Tumours of Soft Tissue and Bone*.

[14] Huang D, Sumegi J, Reith JD (2009). MGC3032-MKL2 is the resulting fusion oncogene of (t11;16)(q13;p13) in chondroid lipoma. *Modern Pathology*.

